# ENGAGING OLDER ADULTS IN HEALTH PROMOTION: PILOT STUDY OF TEAM GAMEPLAY OF AN EDUCATIONAL EXERGAME IN A SENIOR CENTER

**DOI:** 10.1093/geroni/igac059.2696

**Published:** 2022-12-20

**Authors:** Laurie Ruggiero, Elizabeth Orsega-Smith, Amy Nichols, Joshua Varghese, Nancy Getchell, Rachel DeLauder, Reza Koiler, Roghayeh Barmaki

**Affiliations:** University of Delaware, Newark, Delaware, United States; University of Delaware, Newark, Delaware, United States; University of Delaware, Newark, Delaware, United States; Wilmington University, Williamstown, New Jersey, United States; University of Delaware, Newark, Delaware, United States; University of Delaware, Newark, Delaware, United States; University of Delaware, Newark, Delaware, United States; University of Delaware, Newark, Delaware, United States

## Abstract

Digital health games offer one innovative approach to engage older adults to support healthy aging. Multiple reviews have described the positive impact of health games. Limited research has examined multi-focus health games implemented in senior centers. Informed by healthy aging theory and community-engaged methods, our multi-disciplinary team developed/refined an educational exergame with a combined focus on educating about healthy lifestyle behaviors (i.e., physical activity, healthy eating), stimulating cognitive functioning, and engaging movement to support healthy aging. A pilot study (Nf13; mean age = 78, 100% female) examined team gameplay (4 sessions in two weeks) in a senior center. Teams (2–3 members) worked together to answer knowledge, trivia, and cognitive challenge questions and competed for the highest score. A post-gameplay survey asked about acceptability, usability (i.e., adapted System Usability Scale), and perceived game impact. Preliminary results suggest team gameplay was engaging and nearly all (>90%) agreed/strongly agreed that they enjoyed playing with others (i.e., on teams); were comfortable doing the physical movements during gameplay; were satisfied with game educational, trivia, and cognitive questions; enjoyed the social part of team gameplay; would recommend the game to others; and the game increased their knowledge and motivation regarding physical activity and healthy eating. The System Usability Scale was above 70, on average, suggesting above average usability for the game. Findings support use of this educational exergame as an innovative way to engage older adults in health promotion. Presentation will describe game development/refinement, senior center pilot, and implications for future research and senior center translation.

